# Evaluating anti-thymocyte globulin induction doses for better allograft and patient survival in Asian kidney transplant recipients

**DOI:** 10.1038/s41598-023-39353-6

**Published:** 2023-08-02

**Authors:** Ye Eun Shim, Youngmin Ko, Jung Pyo Lee, Jin Seok Jeon, Heungman Jun, Jaeseok Yang, Myoung Soo Kim, Seong Jun Lim, Hye Eun Kwon, Joo Hee Jung, Hyunwook Kwon, Young Hoon Kim, Jungbok Lee, Sung Shin, the Korean Organ Transplantation Registry (KOTRY) study group, Jin Min Kong, Oh Jung Kwon, Deok Gie Kim, Cheol Woong Jung, Yeong Hoon Kim, Joong Kyung Kim, Chan-Duck Kim, Ji Won Min, Sik Lee, Yeon Ho Park, Jae Berm Park, Jung Hwan Park, Jong-Won Park, Tae Hyun Ban, Sang Heon Song, Seung Hwan Song, Ho Sik Shin, Chul Woo Yang, Hye Eun Yoon, Kang Wook Lee, Dong Ryeol Lee, Dong Won Lee, Jieun Oh, Sang-Ho Lee, Su Hyung Lee, Yu Ho Lee, Jeong-Hoon Lee, Kyung Hwan Jeong, Ku Yong Chung, Jong Soo Lee, Ju Man Ki, Dong-Wan Chae, Soo Jin Na Choi, Seungyeup Han, Kyu Ha Huh

**Affiliations:** 1 Division of Kidney and Pancreas Transplantation, Department of Surgery, Asan Medical Center, University of Ulsan College of Medicine, 88, Olympic-ro 43-gil, Songpa-gu, Seoul, 05505 Republic of Korea; 2 Department of Nephrology, SMG-SNU Boramae Medical Center, Seoul, Republic of Korea; 3 Department of Internal Medicine, Soonchunhyang University Seoul Hospital, Seoul, Republic of Korea; 4 Department of Surgery, Korea University Anam Hospital, Seoul, Republic of Korea; 5 Division of Nephrology, Department of Internal Medicine, Yonsei University College of Medicine, Severance Hospital, Seoul, Republic of Korea; 6 Department of Surgery, Yonsei University College of Medicine, Seoul, Republic of Korea; 7 Department of Clinical Epidemiology and Biostatistics, Asan Medical Center, University of Ulsan College of Medicine, 88, Olympic-ro 43-gil, Songpa-gu, Seoul, 05505 Republic of Korea; 8 Department of Nephrology, BHS Hanseo Hospital, Busan, South Korea; 9 Department of Surgery, College of Medicine, Han Yang University, Seoul, South Korea; 10 Department of Surgery, Yonsei University Wonju College of Medicine, Wonju Severance Christian Hospital, Wonju, South Korea; 11 Department of Surgery, Korea University Anam Hospital, Seoul, South Korea; 12 Department of Internal Medicine, Inje University Busan Paik Hospital, Busan, South Korea; 13 Department of Internal Medicine, Bongseng Memorial Hospital, Busan, South Korea; 14 Department of Internal Medicine, School of Medicine, Kyungpook National University Hospital, Daegu, South Korea; 15 Division of Nephrology, Department of Internal Medicine, Bucheon St. Mary’s Hospital, Bucheon, South Korea; 16 Department of Internal Medicine, Jeonbuk National University Hospital, Jeonju, South Korea; 17 Department of Surgery, Gil Medical Center, Gachon University College of Medicine, Seoul, South Korea; 18 Department of Surgery, Samsung Medical Center, Sungkyunkwan University School of Medicine, Seoul, South Korea; 19 Department of Nephrology, Konkuk University School of Medicine, Seoul, South Korea; 20 Department of Nephrology, Yeungnam University Hospital, Daegu, South Korea; 21 Division of Nephrology, Department of Internal Medicine, Eunpyeong St. Mary’s Hospital, Seoul, South Korea; 22 Department of Internal Medicine, Pusan National University Hospital, Busan, South Korea; 23 Department of Surgery, Ewha Womans University Seoul Hospital, Seoul, South Korea; 24 Division of Nephrology, Department of Internal Medicine, Kosin University College of Medicine, Busan, South Korea; 25 Division of Nephrology, Department of Internal Medicine, Seoul St. Mary’s Hospital, Seoul, South Korea; 26 Department of Internal Medicine, Incheon St. Mary’s Hospital, College of Medicine, The Catholic University of Korea College of Medicine, Seoul, South Korea; 27 Department of Nephrology, Chungnam National University Hospital, Daejeon, South Korea; 28 Division of Nephrology, Department of Internal Medicine, Maryknoll Medical Center, Hamilton, Australia; 29 Division of Nephrology, Department of Internal Medicine, Pusan National University School of Medicine, Yangsan, South Korea; 30 Department of Internal Medicine, Kangdong Sacred Heart Hospital, Hallym University College of Medicine, Chuncheon, South Korea; 31 Department of Nephrology, Kyung Hee University Hospital at Gangdong, Seoul, South Korea; 32 Department of Surgery, Ajou University School of Medicine, Suwon, South Korea; 33 Division of Nephrology, Department of Internal Medicine, CHA Bundang Medical Center, CHA University, Seongnam, Korea; 34 Department of Surgery, Myongji Hospital, Goyang, South Korea; 35 Department of Internal Medicine, Kyung Hee University College of Medicine, Seoul, South Korea; 36 Department of Surgery, Ewha Womans University Mokdong Hospital, Seoul, South Korea; 37 Department of Surgery, Ulsan University Hospital, Ulsan, South Korea; 38 Department of Surgery, Gangnam Severance Hospital, Yonsei University College of Medicine, Seoul, South Korea; 39 Division of Nephrology, Seoul National University Bundang Hospital, Seongnam, South Korea; 40 Department of Surgery, Chonnam National University Medical School, Gwangju, South Korea; 41 Department of Internal Medicine, Keimyung University School of Medicine, Daegu, Korea

**Keywords:** Allotransplantation, Renal replacement therapy

## Abstract

Anti-thymocyte globulin (ATG) is currently the most widely prescribed induction regimen for preventing acute rejection after solid organ transplantation. However, the optimal dose of ATG induction regimen in Asian kidney recipients is unclear. Using the Korean Organ Transplantation Registry, we performed a retrospective cohort study of 4579 adult patients who received renal transplantation in South Korea and divided them into three groups according to the induction regimen: basiliximab group (n = 3655), low-dose ATG group (≤ 4.5 mg/kg; n = 467), and high-dose ATG group (> 4.5 mg/kg; n = 457). We applied the Toolkit for Weighting and Analysis of Nonequivalent Groups (TWANG) package to generate high-quality propensity score weights for intergroup comparisons. During four-year follow-ups, the high-dose ATG group had the highest biopsy-proven acute rejection rate (basiliximab 20.8% vs. low-dose ATG 22.4% vs. high-dose ATG 25.6%; P < 0.001). However, the rates of overall graft failure (4.0% vs. 5.0% vs. 2.6%; P < 0.001) and mortality (1.7% vs. 2.8% vs. 1.0%; P < 0.001) were the lowest in the high-dose ATG group. Our results show that high-dose ATG induction (> 4.5 mg/kg) was superior to basiliximab and low-dose ATG induction in terms of graft and patient survival in Asian patients undergoing kidney transplant.

## Introduction

Anti-thymocyte globulin (ATG) is currently the most widely prescribed induction regimen for solid organ transplantation globally. Along with T-cell depletion, ATG is known to be involved in the modulation of adhesion and cell surface molecules that regulate leukocyte-endothelial interaction and T-cell function^1–4^. ATG was shown to be superior to interleukin 2 antagonist for the prevention of acute rejection after kidney transplantation (KT)^5,6^, although post-transplant complications such as infection and malignancy are more common^7–9^.

According to previous reports, the proper doses of ATG as an induction regimen in kidney transplantation ranged from 1.5 to 7.5 mg/kg^1,10–13^. Over a few decades, there has been a trend toward lowering the ATG dose considering the balance between efficacy and safety^14^. A retrospective study reported that the incidence of early acute rejection was higher if the total ATG dose was less than 6 mg/kg^15^. However, favorable outcomes with lower doses have also been reported. Gurk-Turner et al. showed that total ATG doses ≤ 7.5 mg/kg are safe and effective even in high-risk kidney transplant recipients compared with higher doses^12^. Klem et al. suggested that induction with a total ATG dose of 4.5 or 6.0 mg/kg (1.5 mg/kg/dose) was enough to prevent acute rejection even in recipients with high risk^10^.

Recently, the efficacy and immunophenotyping of ATG doses lower than 4.5 mg/kg have also been evaluated. A randomized pilot study suggested that low-dose ATG (2.25 mg/kg) was efficacious in preventing acute rejection and depleting T cells with lower infectious complications^3^. According to Kho et al., T cells in the low-dose (3.0 mg/kg) and ultra-low-dose (1.5 mg/kg) ATG groups returned to control values earlier than the standard dose (6.0 mg/kg) group, although there was no significant differences in clinical outcomes^11^. However, the existing studies on different dosing strategies for ATG are limited due to the small number of patients, predominance of Caucasians, and the exclusion of sensitized patients. Multicenter studies are needed to determine the optimal dose of anti-thymocyte globulin for improved outcomes after KT in different ethnicities.

The aim of this study was to compare the clinical outcomes of KT in an Asian population according to the induction regimen (basiliximab, low-dose, and high-dose ATG) using the nationwide Korean Organ Transplantation Registry (KOTRY) database.

## Results

### Baseline characteristics

The clinical characteristics of the recipients and donors are shown in Table 1 with adjustment by propensity score weighting (TWANG package, Fig. S1). Recipients in the basiliximab group were significantly younger than those in the low-dose ATG and the high-dose ATG groups (48.9 ± 11.6 vs. 51.4 ± 10.7 vs. 51.1 ± 10.8 years, P < 0.001). The high-dose ATG group had the highest proportion of female recipients (37.3% vs. 44.5% vs. 56.5%, P < 0.001) and the lowest body mass index (23.2 ± 3.6 vs. 23.5 ± 3.4 vs. 22.6 ± 3.1 kg/m^2^, P < 0.001). The basiliximab group had the highest proportion of those who underwent preemptive KT (16.4% vs. 9.9% vs. 10.7%, P < 0.001) and ABO-incompatible KT (17.8% vs. 15.2% vs. 12.5%, P = 0.009) and the lowest proportion of those with a history of previous transplant (5.4% vs. 14.4% vs. 16.2%, P < 0.001). The basiliximab group also had the lowest numbers of ABDR mismatch (3.91 ± 1.17 vs. 4.18 ± 1.16 vs. 4.17 ± 1.13, P < 0.001) and DR mismatch (1.29 ± 0.50 vs. 1.40 ± 0.52 vs. 1.39 ± 0.51, P < 0.001). Compared with the ATG groups, the basiliximab group had lower proportions of deceased donors (32.6% vs. 57.6% vs. 54.1%, P < 0.001) and donors with hypertension (14.1% vs. 20.1% vs. 20.1%, P < 0.001) and a higher proportion of current smokers (67.1% vs. 57.8% vs. 59.1%, P < 0.001). Lastly, the basiliximab group had the lowest level of serum creatinine before donation (0.95 ± 0.70 vs. 1.41 ± 1.32 vs. 1.46 ± 1.39 mg/dL, P < 0.001).

**Table 1:** Basal characteristics of the study patients according to the induction regimen.

Variables	Unadjusted					Adjusted			
	Basiliximab	Low dose ATG	High dose ATG	P-value	SMD	Basiliximab	Low dose ATG	High dose ATG	SMD
	(n = 3655)	(n = 467)	(n = 457)			(n = 3655)	(n = 467)	(n = 457)	
Recipient characteristics									
Age, years	48.9 ± 11.6	51.4 ± 10.7	51.1 ± 10.8	< 0.001	0.222	49.2 ± 12.8	49.8 ± 29.9	49.4 ± 31.3	0.074
Female sex	1362 (37.3)	208 (44.5)	258 (56.5)	< 0.001	0.392	(39.1)	(39.7)	(42.3)	0.116
Body mass index, kg/m^2^	23.2 ± 3.6	23.5 ± 3.4	22.6 ± 3.1	< 0.001	0.267	23.1 ± 3.9	23.8 ± 10.1	22.9 ± 8.8	0.066
Hypertension	3303 (90.4)	412 (88.2)	400 (87.5)	0.076	0.094	(90.3)	(86.8)	(87.9)	0.055
Diabetes mellitus	1119 (30.6)	152 (32.6)	137 (30.0)	0.648	0.058	(30.7)	(29.7)	(32.3)	0.060
Primary cause of ESRD									
Diabetes mellitus	885 (24.2)	121 (25.9)	109 (23.9)	0.577	0.055	(23.9)	(24.3)	(26.2)	0.090
Hypertension	542 (14.8)	69 (14.8)	81 (17.7)			(15.1)	(15.6)	(21.2)	
Glomerulonephritis	1192 (32.6)	165 (35.5)	151 (33.0)			(32.3)	(37.0)	(30.7)	
Tubulointerstitial nephritis	14 (0.4)	2 (0.4)	2 (0.4)			(0.4)	(0.4)	(0.7)	
Polycystic kidney disease	182 (5.0)	20 (4.3)	24 (5.3)			(5.0)	(4.4)	(4.5)	
Hereditary kidney disease except PCKD	52 (1.4)	3 (0.6)	4 (0.9)			(1.4)	(0.6)	(0.8)	
Obstructive uropathy	17 (0.5)	0 (0)	0 (0)			(0.5)	(0.0)	(0.0)	
Others	37 (1.0)	2 (0.4)	4 (0.9)			(1.1)	(0.4)	(0.9)	
Unknown	734 (20.1)	85 (18.2)	82 (17.9)			(20.4)	(17.4)	(15.0)	
Previous transplant	198 (5.4)	67 (14.4)	74 (16.2)	< 0.001	0.412	(6.9)	(7.9)	(9.3)	0.108
Preemptive transplant	600 (16.4)	46 (9.9)	49 (10.7)	< 0.001	0.183	(15.3)	(16.0)	(15.8)	0.034
ABO-incompatible KT	651 (17.8)	71 (15.2)	57 (12.5)	0.009	0.142	(16.9)	(18.8)	(17.9)	0.042
ABDR mismatch	3.91 ± 1.17	4.18 ± 1.16	4.17 ± 1.13	< 0.001	0.234	3.94 ± 1.30	3.98 ± 3.32	4.02 ± 3.11	0.073
DR mismatch	1.29 ± 0.50	1.40 ± 0.52	1.39 ± 0.51	< 0.001	0.225	1.30 ± 0.56	1.32 ± 1.39	1.34 ± 1.33	0.079
Panel reactive antibody, %									
PRA1	9.2 ± 20.8	16.2 ± 27.0	22.0 ± 31.2	< 0.001	0.210	10.5 ± 25.0	13.9 ± 66.1	14.8 ± 71.0	0.097
PRA2	9.2 ± 21.5	15.2 ± 28.0	23.3 ± 32.7	< 0.001	0.201	10.5 ± 25.8	12.6 ± 67.1	14.80 ± 74.0	0.104
Pre-transplant DSA	249 (6.8)	89 (19.1)	143 (31.3)	< 0.001	0.056	(9.2)	(13.4)	(13.8)	0.082
Missing	1325 (36.3)	147 (31.5)	104 (22.8)			(35.1)	(35.1)	(32.1)	
Desensitization	779 (21.3)	133 (28.5)	160 (35.0)	< 0.001	0.322	(23.0)	(26.0)	(24.6)	0.070
Calcineurin inhibitor	3641 (99.6)	465 (99.6)	456 (99.8)	0.843	0.034	(99.6)	(99.7)	(99.8)	0.031
Tacrolimus	3524 (96.4)	456 (97.6)	454 (99.3)	0.002		(96.5)	(97.0)	(98.6)	
Cyclosporine	117 (3.2)	9 (1.9)	2 (0.4)	0.002		(3.2)	(2.7)	(1.2)	
Mycophenolate mofetil	3454 (94.5)	453 (97.0)	441 (96.5)	0.019	0.114	(94.7)	(98.3)	(98.3)	0.164
Sirolimus or everolimus	15 (0.4)	1 (0.2)	2 (0.4))	0.913	0.036	(0.4)	(0.1)	(0.3)	0.054
Steroid	3623 (99.1)	460 (98.5)	439 (96.1)	< 0.001	0.276	(99.0)	(97.2)	(98.6)	0.162
Donor characteristics									
Age, years	47.1 ± 12.8	48.9 ± 13.4	47.7 ± 13.4	0.018	0.135	47.3 ± 14.3	47.6 ± 34.1	48.1 ± 35.0	0.067
Female sex	1846 (50.5)	183 (39.2)	170 (37.2)	< 0.001	0.266	(48.5)	(46.9)	(47.8)	0.042
Body mass index, kg/m^2^	23.9 ± 3.3	24.0 ± 3.5	23.8 ± 3.6	0.521	0.074	23.9 ± 3.7	23.8 ± 8.8	24.0 ± 8.9	0.056
Relation to the recipient									
Deceased donor	1193 (32.6)	269 (57.6)	247 (54.1)	< 0.001	0.388	(36.4)	(38.0)	(40.4)	0.082
Living, related	1434 (39.2)	94 (20.1)	113 (24.7)			(36.7)	(32.4)	(33.9)	
Living, unrelated	1028 (28.1)	104 (22.3)	97 (21.2)			(26.9)	(29.7)	(26.3)	
CRRT	51 (1.4)	34 (7.3)	18 (3.9)	< 0.001	0.467	(1.7)	(3.5)	(2.3)	0.119
Missing	2528 (69.2)	207 (44.3)	218 (47.7)			(65.5)	(64.0)	(60.7)	
Current smoker	2452 (67.1)	270 (57.8)	270 (59.1)	< 0.001	0.195	(65.7)	(66.1)	(65.4)	0.032
Hypertension	515 (14.1)	94 (20.1)	92 (20.1)	< 0.001	0.206	(14.9)	(17.2)	(18.0)	
Serum creatinine, mg/dL	0.95 ± 0.70	1.41 ± 1.32	1.46 ± 1.39	< 0.001	0.569	1.03 ± 0.94	1.04 ± 2.36	1.07 ± 2.46	0.073
Cold ischemic time									
0–6 h	2812 (76.9)	341 (73.0)	316 (69.2)	< 0.001	0.137	(76.0)	(78.2)	(74.5)	0.058
6–12 h	192 (5.3)	47 (10.1)	41 (9.0)			(6.0)	(570.0)	(6.4)	
> 12 h	6 (0.2)	5 (1.1)	2 (0.4)			(0.2)	(0.5)	(0.2)	
Missing	645 (17.7)	74 (15.9)	98 (21.4)			(17.8)	(1555.0)	(19.0)	

Values are mean ± standard deviation or n (%).

*SMD* standardized mean difference, *ESRD* end-stage renal disease, *PCKD* polycystic kidney disease, *PRA* panel reactive antibody, *DSA* donor-specific antigen, *CRRT* continuous renal replacement therapy.

### Clinical outcomes

**Figure 1:**
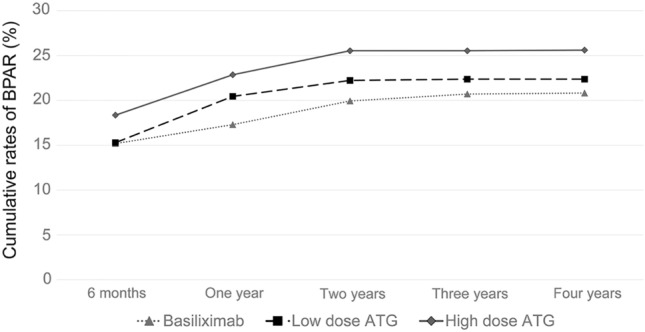
Cumulative rates of biopsy-proven acute rejection.

A total of 1947 kidney transplant recipients had kidney allograft biopsies due to an elevated serum creatinine (n = 773), aggravated proteinuria (n = 40), scheduled protocol (n = 1081), and other reasons (n = 53). Cumulative incidences of biopsy-proven acute rejection (BPAR) were significantly higher in the high-dose ATG group than in the other groups during a four-year follow-up period, although the increase nearly plateaued two years after transplantation (Table 2) (Fig. 1). The rates of acute antibody-mediated rejection (ABMR) and chronic ABMR were the highest in the high-dose ATG group. In contrast, acute T-cell mediated rejection (TCMR) within 1 year post-transplantation was the highest in the low-dose ATG (Table 3).

**Table 2:** Clinical outcomes according to the induction regimen.

Adjusted						Bonfferoni adjusted p-value		
Basiliximab		Low-dose ATG	High-dose ATG	p-value 1	p-value 2	B vs. L	B vs. H	L vs. H
Biopsy-proven acute rejection (%)								
6 months	15.2	15.3	18.4	0.174	< 0.001	1.000	< 0.001	0.002
1 year	17.3	20.5	22.9	< 0.001	< 0.001	< 0.001	< 0.001	0.043
2 years	19.9	22.2	25.5	< 0.001	< 0.001	0.036	< 0.001	0.004
3 years	20.7	22.4	25.5		< 0.001	0.212	< 0.001	0.006
4 years	20.8	22.4	25.6		< 0.001	0.275	< 0.001	0.005
Overall graft failure (%)								
6 months	2.0	2.8	1.4	0.308	< 0.001	0.053	0.176	< 0.001
1 year	2.7	3.6	1.8	< 0.001	< 0.001	0.093	0.014	< 0.001
2 years	3.3	4.6	2.5	0.150	< 0.001	0.006	0.137	< 0.001
3 years	3.8	5.0	2.6		< 0.001	0.028	0.007	< 0.001
4 years	4.0	5.0	2.6		< 0.001	0.096	0.002	< 0.001
Death-censored graft failure (%)								
6 months	1.1	0.7	1.0	0.907	0.167	0.206	1.000	0.371
1 year	1.5	1.4	1.1	0.060	0.363	1.000	0.505	0.800
2 years	1.9	2.2	1.6	0.073	0.157	0.745	1.000	0.171
3 years	2.3	2.2	1.7		0.121	1.000	0.156	0.262
4 years	2.4	2.2	1.7		0.080	1.000	0.084	0.262
Mortality (%)								
6 months	0.9	2.1	0.4	0.052	< 0.001	< 0.001	0.019	< 0.001
1 year	1.2	2.1	0.6	0.017	< 0.001	0.005	0.020	< 0.001
2 years	1.4	2.4	0.9	0.023	< 0.001	0.004	0.117	< 0.001
3 years	1.6	2.8	1.0		< 0.001	< 0.001	0.053	< 0.001
4 years	1.7	2.8	1.0		< 0.001	0.002	0.019	< 0.001
Delayed graft function (%)								
	3.40	2.95	4.28		0.009	0.7831	0.123	0.009

p-value1: generalized linear mixed model (group effect, time effect and interaction of group & time).

p-valu2: group comparsion at each time point.

*B* basiliximab, *L* low-dose ATG, *H* high-dose ATG.

**Table 3:** Biopsy proven acute rejection according to the induction regimen.

Adjusted						Bonfferoni adjusted p-value		
Basiliximab		Low-dose ATG	High-dose ATG	p-value 1	p-value 2	B vs. L	B vs. H	L vs. H
Acute T cell-mediated rejection (%)								
6 months	6.21	8.02	7.14	0.100	0.007	0.005	0.301	0.481
1 year	7.11	11.40	8.47	< 0.001	< 0.001	< 0.001	0.072	< 0.001
2 years	7.86	12.16	11.11	0.046	< 0.001	< 0.001	< 0.001	0.505
3 years	8.03	12.21	11.11		< 0.001	< 0.001	< 0.001	0.452
4 years	8.09	12.21	11.11		< 0.001	< 0.001	< 0.001	0.452
Acute antibody-mediated rejection (%)								
6 months	2.60	2.57	4.59	0.442	< 0.001	< 0.999	< 0.001	< 0.001
1 year	3.10	3.11	4.83	< 0.001	< 0.001	< 0.999	< 0.001	< 0.001
2 years	3.53	3.30	5.08	0.008	0.001	< 0.999	0.002	< 0.001
3 years	3.99	3.30	5.08		< 0.001	0.313	0.057	< 0.001
4 years	4.01	3.30	5.16		< 0.001	0.278	0.045	< 0.001
Chronic active T cell-mediated rejection (%)								
6 months	0.24	0.12	0.67	0.345	< 0.001	0.572	0.013	< 0.001
1 year	0.49	0.19	0.88	0.243	< 0.001	0.062	0.105	< 0.001
2 years	0.65	0.19	1.58	< 0.001	< 0.001	0.005	< 0.001	< 0.001
3 years	0.74	0.19	1.58		< 0.001	0.001	0.001	< 0.001
4 years	0.74	0.19	1.58		< 0.001	0.001	0.001	< 0.001
Chronic active antibody-mediated rejection (%)								
6 months	0.11	0.00	0.99	0.311	< 0.001	0.138	< 0.001	< 0.001
1 year	0.20	0.40	0.99	0.458	< 0.001	0.290	< 0.001	0.008
2 years	0.39	0.40	0.99	0.040	< 0.001	> 0.999	0.003	0.008
3 years	0.57	0.40	0.99		0.006	0.873	0.086	0.008
4 years	0.57	0.40	0.99		0.006	0.873	0.086	0.008

p-value1: generalized linear mixed model (group effect, time effect and interaction of group & time).

p-valu2: group comparsion at each time point.

*B* basiliximab, *L* low-dose ATG, *H* high-dose ATG.

**Figure 2:**
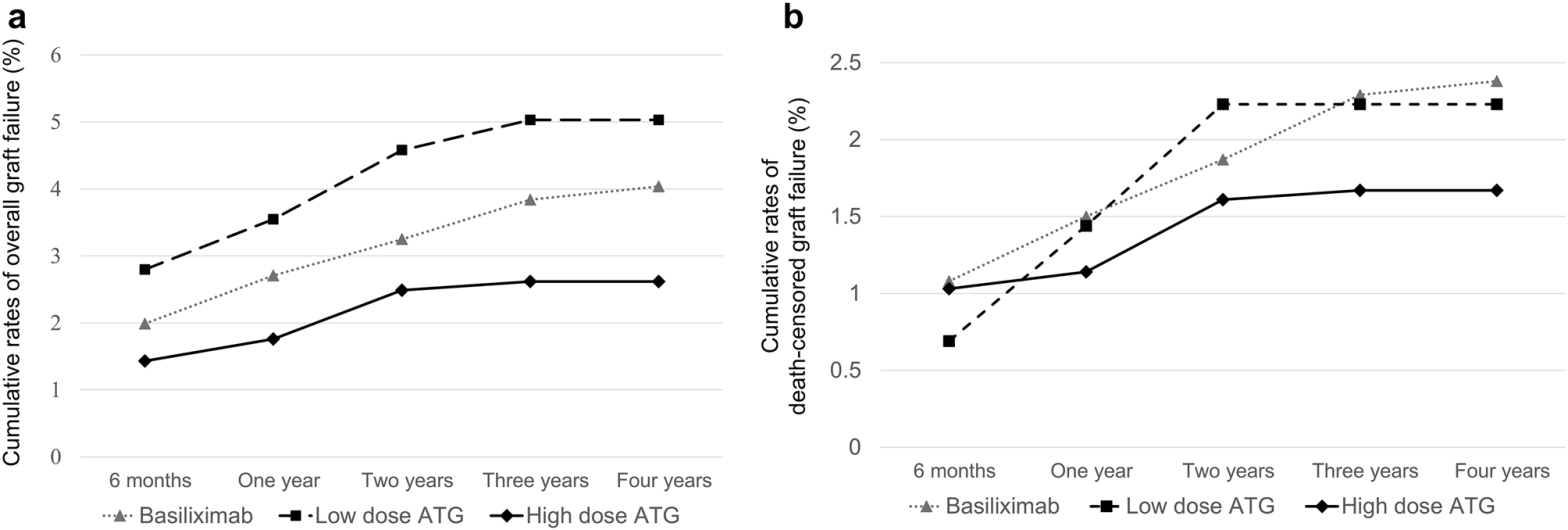
Cumulative rates of overall graft failure (**a**) and death-censored graft failure (**b**).

**Figure 3:**
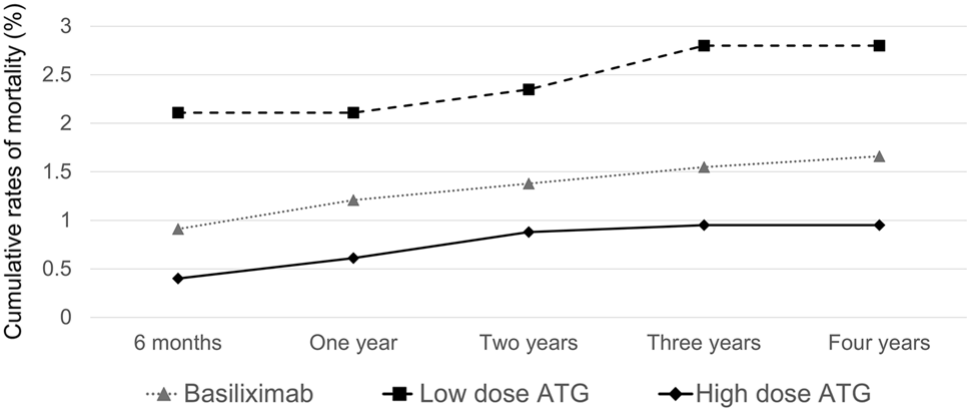
Cumulative rates of mortality.

**Figure 4:**
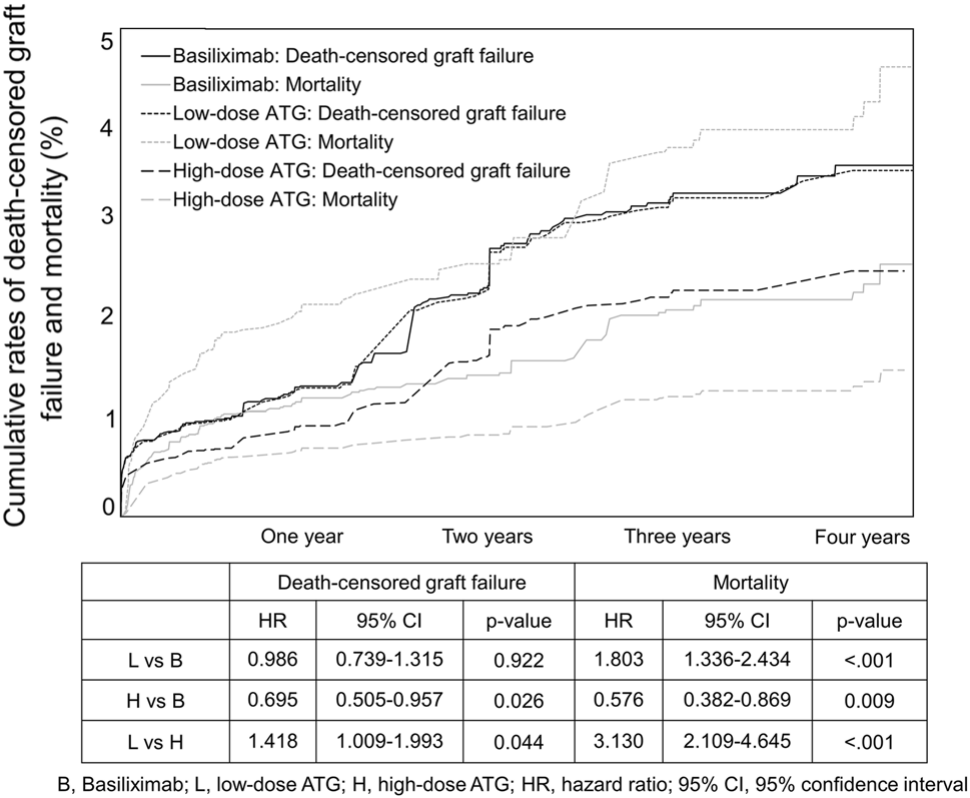
The fine-gray subdistribution hazard ratio analysis for death-censored graft failure and mortality.

The rate of delayed graft function was the highest in the high-dose ATG group (3.40% vs. 2.95% vs. 4.28%, P = 0.009) (Table 2). In contrast, the cumulative rate of overall graft failure was the lowest in the high-dose ATG group (Fig. 2a). Also, the rate of death-censored graft failure (DCGF) was the lowest in the high-dose ATG group, albeit without statistical significance (P = 0.08; Fig. 2b). The cumulative rate of mortality was the lowest in the high-dose ATG group and the highest in the low-dose ATG group (Fig. 3). Since significant differences in the mortality among groups is identified, the graft survival was re-analyzed using Fine-Gray Subdistribution Hazard Ratio (HR) analysis (Fig. 4). The results of the Fine-Gray Subdistribution HR analysis for DCGF and mortality show a significant increase in mortality risk for the low-dose ATG group compared to both the Basiliximab and high-dose ATG groups, with the highest risk observed when comparing low-dose ATG to high-dose ATG. Additionally, the risk of DCGF was significantly lower in the high-dose ATG group compared to Basiliximab, while there was no significant difference between low-dose ATG and Basiliximab. These findings suggest that patients receiving low-dose ATG may have a higher risk of mortality, and that high-dose ATG may provide better protection against DCGF compared to Basiliximab.

**Figure 5:**
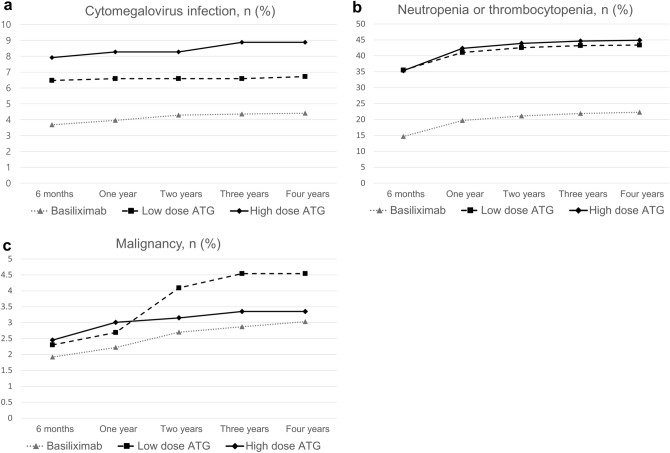
Cumulative rates of complication after transplantation. Cytomegalovirus infection (**a**), neutropenia or thrombocytopenia (**b**), and malignancy (**c**).

Cytomegalovirus infection was the most common in the high-dose ATG group (Fig. 5a), and hematologic abnormalities including neutropenia and thrombocytopenia were higher in the ATG groups than in the basiliximab group (Fig. 5b). Although there was no significant difference in the incidence of post-transplant malignancy until two years after transplantation, the incidence in the low-dose ATG group was higher thereafter (Fig. 5c).

Furthermore, we evaluated clinical outcomes based on induction regimens used in patients considered at high immunological risk. High immunological risk patients were defined as those who underwent desensitization before kidney transplantation due to the presence of pre-transplant donor-specific antibodies, ABO-incompatible KT, or human leukocyte antigen (HLA)-incompatible KT. Out of the 4579 enrolled recipients, 1072 were classified as high-risk immunologically. Among them, 779 recipients were administered basiliximab, while 133 and 160 recipients received low- and high-dose ATG, respectively. Over a four-year follow-up period, the cumulative incidences of BPAR were significantly higher in the low- and high-dose ATG groups compared to the basiliximab group, but no significant difference was observed between the low- and high-dose ATG groups (Table 4). Conversely, the cumulative rates of overall graft failure and mortality were significantly lower in the high-dose ATG group compared to the low-dose ATG group, while no significant difference was found in the cumulative rate of DCGF between the groups.

**Table 4:** Clinical outcomes according to the induction regimen in immunologic high-risk patients.

Adjusted						Bonfferoni adjusted p-value		
Basiliximab		Low-dose ATG	High-dose ATG	p-value 1	p-value 2	B vs. L	B vs. H	L vs. H
Biopsy-proven acute rejection (%)								
6 months	16.8	22.8	25.7	0.025	< 0.001	0.002	< 0.001	0.289
1 year	18.6	29.1	29.1	< 0.001	< 0.001	< 0.001	< 0.001	0.989
2 years	20.7	32.3	31.5	0.256	< 0.001	< 0.001	< 0.001	0.661
3 years	21.3	32.4	31.5		< 0.001	< 0.001	< 0.001	0.608
4 years	21.4	32.4	31.7		< 0.001	< 0.001	< 0.001	0.608
Overall graft failure (%)								
6 months	1.6	2.3	0.7	0.576	0.010	0.782	0.175	0.006
1 year	2.5	2.3	0.8	< 0.001	0.007	1.000	0.008	0.015
2 years	2.9	5.0	3.0	< 0.001	0.014	0.044	1.000	0.032
3 years	3.0	5.0	3.0		0.019	0.066	1.000	0.032
4 years	3.0	5.0	3.0		0.019	0.066	1.000	0.032
Death-censored graft failure (%)								
6 months	0.8	0.6	0.74	0.951	0.926	1.000	1.000	1.000
1 year	1.3	0.6	0.7	0.367	0.185	0.291	0.517	1.000
2 years	1.5	3.1	2.1	0.047	0.043	0.043	0.726	0.481
3 years	1.6	3.1	2.1		0.066	0.074	1.000	0.481
4 years	1.6	3.1	2.1		0.066	0.074	1.000	0.481
Mortality (%)								
6 months	0.9	1.7	0.0	0.432	< 0.001	0.293	0.005	< 0.001
1 year	1.5	1.7	0.1	0.042	< 0.001	0.951	0.005	< 0.001
2 years	1.4	1.9	0.9	< 0.001	0.130	1.000	0.807	0.043
3 years	1.4	1.9	0.9		0.130	1.000	0.807	0.043
4 years	1.4	1.9	0.9		0.130	1.000	0.807	0.043

p-value 1: generalized linear mixed model (group effect, time effect and interaction of group and time).

p-value 2: group comparsion at each time point.

*B* basiliximab, *L* low-dose ATG, *H* high-dose ATG.

## Discussion

Using the KOTRY database, we found that the mean, median, 25th percentile, and 75th percentile of the ATG dose in Korean KT recipients were 4.85 mg/kg, 4.50 mg/kg, 4.26 mg/kg, and 5.45 mg/kg, respectively. We also found that high-dose ATG induction (> 4.5 mg/kg) showed superior outcomes in terms of graft and patient survival compared with basiliximab and low-dose ATG (< 4.5 mg/kg), although BPAR was the most common in the high-dose ATG group. To our knowledge, this is the first study to examine the currently used dose of ATG as an induction regimen and to compare the clinical outcomes according to the doses of ATG using a national kidney transplant database in an Asian population.

Previous randomized controlled trials compared clinical outcomes according to the doses of ATG as an induction regimen, which ranged from 5 to 10 mg/kg^19–25^. On the other hand, recent studies examined the efficacies of lower doses of ATG on the depletion of immune cells as well as clinical outcomes. Kho et al. compared the counts of peripheral immune cells after kidney transplantation among 1.5, 3.0, and 6.0 mg/kg ATG induction groups and showed that only the 6.0 mg/kg ATG induction group had a significantly lower T cell count compared with recipients without an induction regimen^11^. According to one randomized pilot study, 2.25 mg/kg ATG induction with early steroid tapering was effective not only in preventing acute rejection but also in depleting T cells^3^.

A recent study by Mehta et al. showed that patients in the regular dose ATG (≥ 5 mg/kg) group had more favorable outcomes in terms of early (within six months posttransplant) rejection compared with the low-dose (< 5 mg/kg) group^26^. On the other hand, another study by Linhares et al. reported that the 3 mg/kg and 5 mg/kg ATG induction groups did not show significant differences in the incidence of efficacy failure (first BPAR, graft failure, or mortality) and in safety outcomes at twelve months posttransplant^27^; however, the study by Linhares et al. was limited due to its single-center, retrospective design, small number of patients, short follow-up duration, and the fact that only those who underwent repeat kidney transplantation were included.

Even though the incidence of BPAR was the highest in the high-dose ATG group in our study, recipients in this group showed superior outcomes in terms of graft and patient survival. Considering that the high-dose ATG group had the highest incidence of BPAR during the first 6 months, it is likely that the high-dose ATG group had more patients with higher immunologic risk although we tried to create high-quality propensity score weights with the TWANG package. Although it is known that ATG is effective to prevent and treat TCMR, there are several reports that ATG is also effective against ABMR by suppression of natural killer (NK) and follicular helper T cells. Several studies using microarray transcriptomic analysis showed that NK and follicular helper T cells are actively involved in the pathophysiology of ABMR and graft failure after kidney transplantation^28–32^. Recently, we reported the results of a prospective, randomized, pilot study showing that NK cells in recipients with ATG 6.0 mg/kg induction remained suppressed whereas NK cells in those with ATG 6.0 mg/kg induction had recovered to the pre-transplant levels^33^. Therefore, it is considered that those with high-dose ATG induction had superior outcomes in terms of graft failure and mortality in spite of higher incidences of ABMR.

Interestingly, not only the rate of developing malignancy but also the rates of graft failure and mortality were the highest in the low-dose ATG group. Higher incidences of cytomegalovirus infection, neutropenia, or thrombocytopenia in the high-dose ATG group did not have a significant impact on graft failure and mortality. The unique observation of mortality preceding death-censored graft failure in the low-dose ATG group highlights the need for further investigation to understand the underlying factors contributing to this phenomenon.

This study has several limitations. We could not assess the presence of donor-specific antibodies before transplantation, which is necessary for estimating the immunologic risk of each patient. In addition, data on calcineurin inhibitor trough levels were not collected during the follow-up period, and Banff scores for each BPAR were not recorded in the registry. However, we could investigate the incidences of TCMR and ABMR separately. Due to the limited follow-up duration in this nationwide registry, we could not evaluate the long-term clinical outcomes according to the induction regimen. Lastly, the protocols for desensitization and treatment of TCMR and ABMR could not be determined at each center, which could affect graft survival and patient survival after an episode of BPAR.

Nevertheless, it is noteworthy that this study investigated the contemporary practice of KT induction regimens in an Asian population and compared the clinical outcomes according to the induction regimen and ATG dosage using data from a nationwide kidney transplant cohort. Notably, low-dose ATG induction had the worst outcomes even than basiliximab induction in terms of graft survival and patient survival.

In conclusion, our current study in an Asian nationwide cohort showed that high-dose ATG induction (> 4.5 mg/kg) had superior outcomes to basiliximab and low-dose ATG induction in terms of graft and patient survival.

## Materials and methods

### Study population

**Figure 6:**
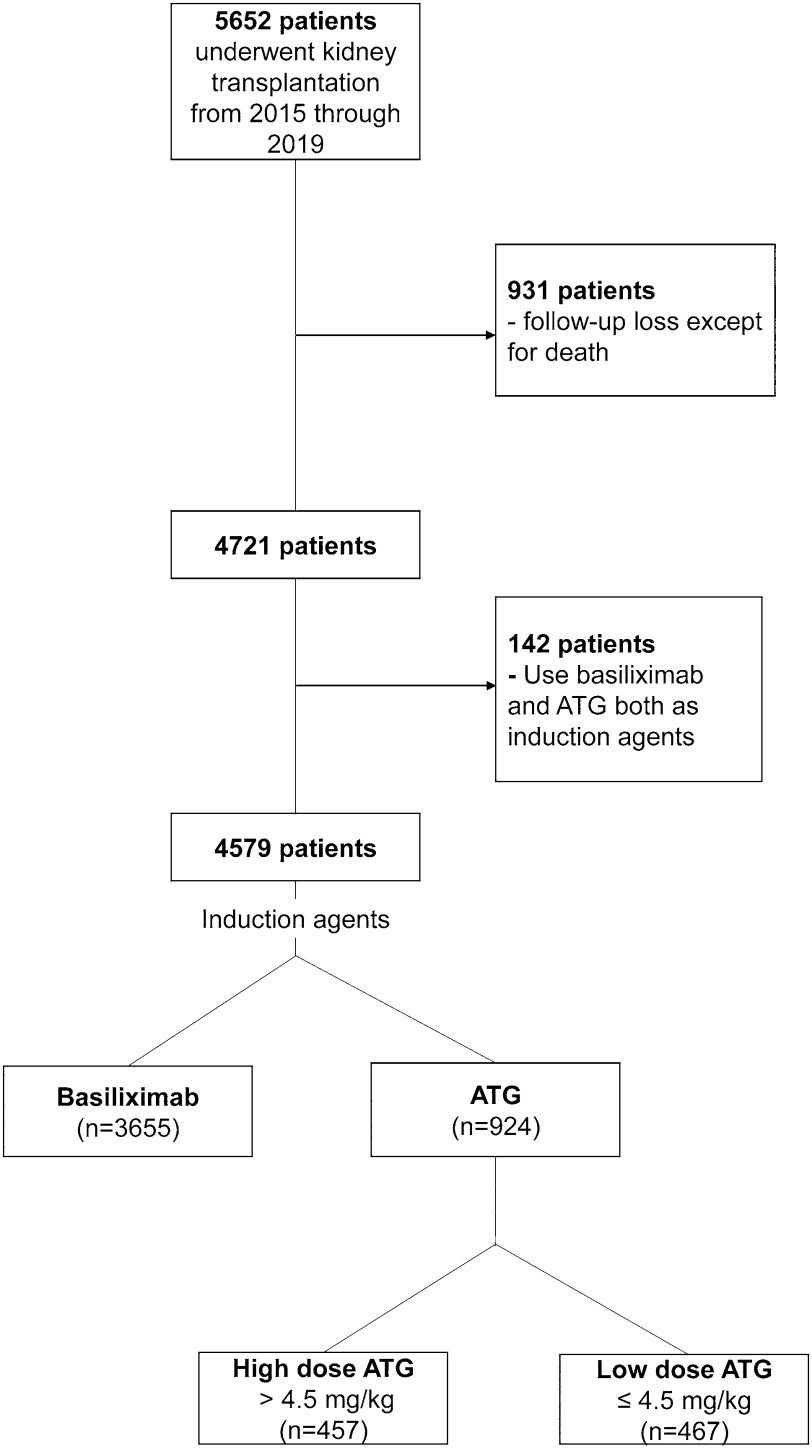
Flow chart of the study population.

**Figure 7:**
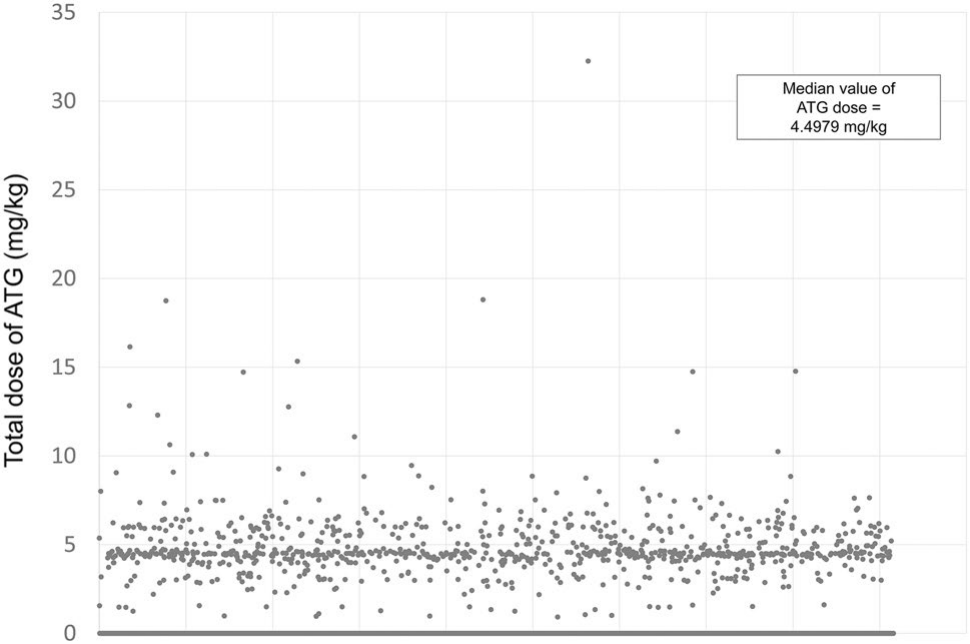
Distribution of the total dose of ATG.

KOTRY is a nationwide transplantation cohort for five solid organs (kidney, liver, heart, lung, and pancreas) consisting of 59 participating centers in South Korea. Longitudinal data collection was initiated in 2014. Details about the design and methods were previously reported^16^. Briefly, KOTRY collects data on demographics, comorbidities, laboratory measurements, concentration and dosage of immunosuppressants including induction and maintenance regimen, allograft rejection, allograft failure, infectious events, cardiovascular complications, malignancies, recipient mortality, donor comorbidity, and outcomes of live donors. The KOTRY dataset includes a total of 5652 kidney transplant recipients from 30 centers between 2015 and 2019, from which 931 were excluded due to follow-up loss except for death and 142 were excluded because both ATG and basiliximab were administered as induction regimen (Fig. 6). Therefore, a total of 4579 recipients were included in this study. Among them, ATG was administered to 924 recipients whereas basiliximab was used for 3655 recipients. The mean, median, 25th percentile, and 75th percentile of the ATG dose were 4.85 mg/kg, 4.50 mg/kg, 4.26 mg/kg, and 5.45 mg/kg, respectively (Fig. 7). Of the 924 recipients with ATG induction, 467 were classified as low-dose ATG group and 457 as high-dose ATG group based on the median value of ATG dose (4.50 mg/kg). Written informed consent was achieved from all recipients and live kidney donors before KT and donor nephrectomy, respectively. This study was approved by the institutional review board of Asan Medical Center (approval number: 2021–0032) and was performed according to the Declaration of Helsinki and the Declaration of Istanbul. No organs/tissues were procured from prisoners.

### Clinical outcomes

The primary outcomes were the rates of BPAR, DCGF, overall graft failure, and mortality. BPAR was diagnosed according to the Banff 2015 criteria^17^. Secondary outcomes were the level of serum creatinine and the incidence of cytomegalovirus infection, bacterial infection, neutropenia or thrombocytopenia, or malignancy. CMV infection was defined as end-organ infection by CMV or persistent detection of CMV viremia (> 3.0 log copies/mL) in polymerase chain reaction tests. Neutropenia was defined as white blood cell count less than 1,500 /uL whereas thrombocytopenia was considered if the platelet count was less than 150,000 /uL. The primary and secondary outcomes were assessed at six months after transplantation and at one-year intervals thereafter.

### Statistical analysis

Continuous variables are presented as mean ± standard deviation (SD). One-way analysis of variance (ANOVA) was used to compare continuous variables among the three groups. Primary and secondary outcomes at each period were compared between groups using ANOVA. Categorical variables are summarized as absolute and relative frequencies, and differences among categorical values were analyzed using the Chi-squared test. In order to create high-quality propensity score weights, the Toolkit for Weighting and Analysis of Nonequivalent Groups (TWANG) package was applied to estimate the probability that a recipient developed primary and secondary outcomes for calculating weights and generalized boosted regression^18^. A total of fifteen recipient characteristics (age, sex, diabetes, previous transplant, ABO-incompatible KT, ABDR mismatch, DR mismatch, and calcineurin inhibitor) and donor characteristics (age, sex, body mass index, relation to the recipient, current smoker, serum creatinine, and cold ischemic time) were included in the weighting algorithm. After the matched samples were identified, generalized linear mixed models were estimated by including all recipient and donor characteristics to adjust for any possible differences among the groups. P values < 0.05 were considered statistically significant. Statistical analyses were performed using R software version 3.1.2 (R Foundation for Statistical Computing, Vienna, Austria).

## Supplementary Information

Supplementary Figure 1.

Supplementary Table 1.

## Data Availability

Raw data were generated at Korea Centers for Disease Control and Prevention Agency. Derived data supporting the findings of this study are available from the corresponding author on request.

## Abbreviations

ABMR: Antibody-mediated rejection
ATG: Anti-thymocyte globulin
BPAR: Biopsy-proven acute rejection
DCGF: Death-censored graft failure
KOTRY: Korean organ transplantation registry
KT: Kidney transplantation
NK: Natural killer
PRA: Panel reactive antibody
TCMR: T-cell mediated rejection
TWANG: Toolkit for weighting and analysis of nonequivalent groups
