# Supplementary material for: Evaluating anti-thymocyte globulin induction doses for better allograft and patient survival in Asian kidney transplant recipients
Source: Sci Rep. 2023 Aug 2;13:12560. doi: 10.1038/s41598-023-39353-6 (PMC10397229; doi:10.1038/s41598-023-39353-6)
Supplement: Supplementary file 1 — Supplementary Figure 1. [file 41598_2023_39353_MOESM1_ESM.pptx]

## Slide 1
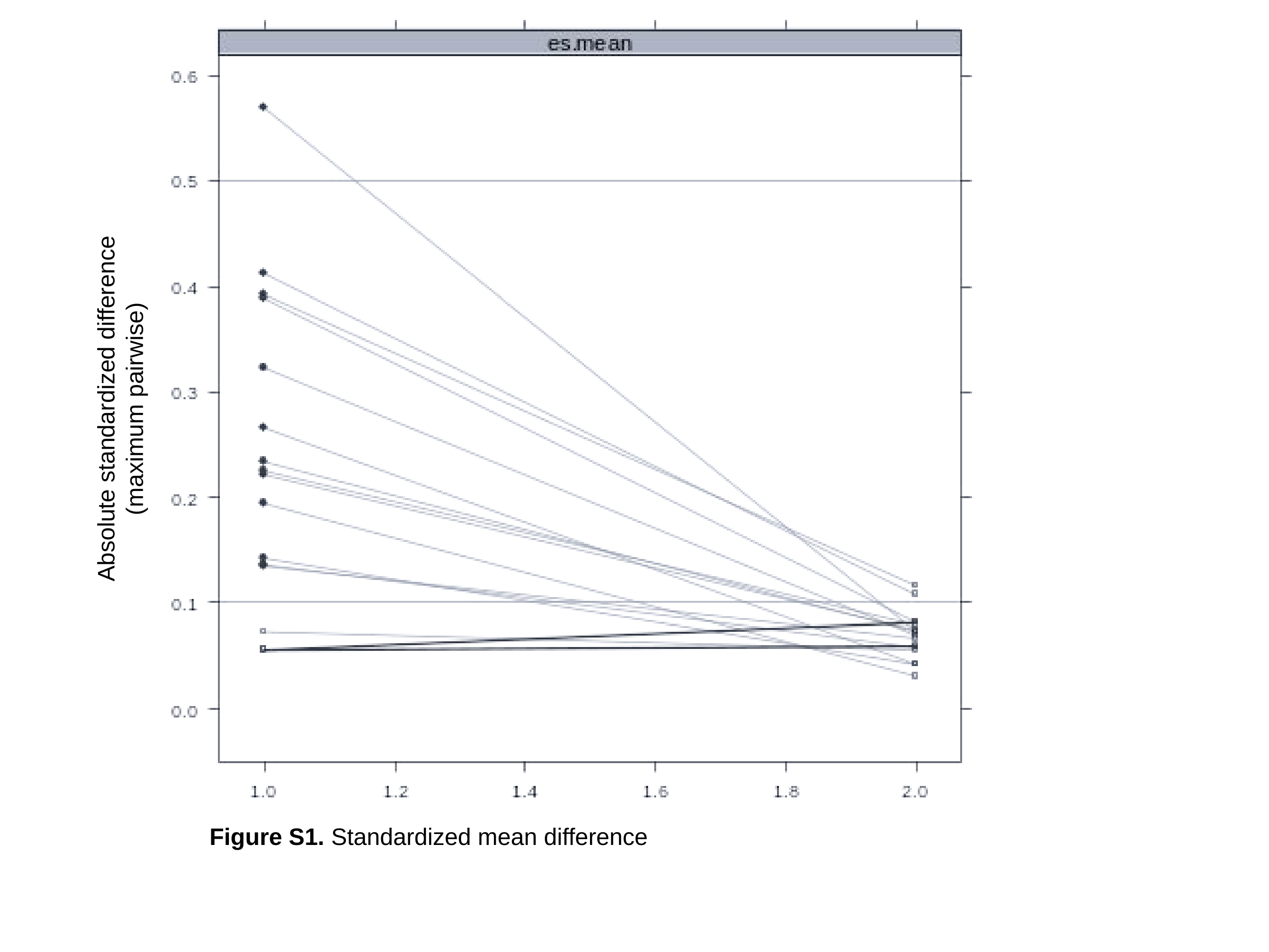

Absolute standardized difference
(maximum pairwise)
Figure S1. Standardized mean difference
