# Supplementary material for: Evaluating anti-thymocyte globulin induction doses for better allograft and patient survival in Asian kidney transplant recipients
Source: Sci Rep. 2023 Aug 2;13:12560. doi: 10.1038/s41598-023-39353-6 (PMC10397229; doi:10.1038/s41598-023-39353-6)
Supplement: Supplementary file 2 — Supplementary Table 1. [file 41598_2023_39353_MOESM2_ESM.docx]

**Evaluating Anti-thymocyte Globulin Induction Doses for Better Allograft and Patient Survival in Asian Kidney Transplant Recipients**

Ye Eun Shim^1^, Youngmin Ko^1^, Jung Pyo Lee^2^ , Jin Seok Jeon^3^, Heungman Jun^4^, Jaeseok Yang^5^, Myoung Soo Kim^6^, Seong Jun Lim^1^, Hye Eun Kwon^1^, Joo Hee Jung^1^, Hyunwook Kwon^1^, Young Hoon Kim^1^, Jungbok Lee^5^, Sung Shin^1^*; and the Korean Organ Transplantation Registry (KOTRY) study group

**Table S1. Basal characteristics of the study patients according to the induction regimen**

| **Variables** | **Basiliximab**  **(n = 3655)** | **Low-dose ATG**  **(n = 467)** | **High-dose ATG**  **(n = 457)** | **P-value** |
| --- | --- | --- | --- | --- |
| **Recipient characteristics** | | | | |
| **Age**, years | 48.9±11.6 | 51.40±10.7 | 51.1±10.8 | <0.001 |
| **Female sex** | 1362 (37.3) | 208 (44.5) | 258 (56.5) | <0.001 |
| **Body mass index**, kg/m^2^ | 23.16 (3.6) | 23.49 (3.4) | 22.55 (3.1) | <0.001 |
| **Hypertension** | 3303 (90.4) | 412 (88.2) | 400 (87.5) | 0.076 |
| **Diabetes mellitus** | 1119 (30.6) | 152 (32.6) | 137 (30.0) | 0.648 |
| **Primary cause of ESRD** |  |  |  | 0.577 |
| Diabetes mellitus | 885 (24.2) | 121 (25.9) | 109 (23.9) |  |
| Hypertension | 542 (14.8) | 69 (14.8) | 81 (17.7) |  |
| Glomerulonephritis | 1192 (32.6) | 165 (35.5) | 151 (33.0) |  |
| Tubulointerstitial nephritis | 14 (0.4) | 2 (0.4) | 2 (0.4) |  |
| Polycystic kidney disease | 182 (5.0) | 20 (4.3) | 24 (5.3) |  |
| Hereditary kidney disease except PCKD | 52 (1.4) | 3 (0.6) | 4 (0.9) |  |
| Obstructive uropathy | 17 (0.5) | 0 (0) | 0 (0) |  |
| Others | 37 (1.0) | 2 (0.4) | 4 (0.9) |  |
| Unknown | 734 (20.1) | 85 (18.2) | 82 (17.9) |  |
| **Previous transplant** | 198 (5.4) | 67 (14.4) | 74 (16.2) | <0.001 |
| **Preemptive transplant** | 600 (16.4) | 46 (9.9) | 49 (10.7) | <0.001 |
| **ABO-incompatible KT** | 651 (17.8) | 71 (15.2) | 57 (12.5) | 0.009 |
| **ABDR mismatch** | 3.91±1.17 | 4.18±1.16 | 4.17±1.13 | <0.001 |
| **DR mismatch** | 1.29±0.50 | 1.40±0.52 | 1.39±0.51 | <0.001 |
| **Panel reactive antibody,** % |  |  |  |  |
| PRA1 | 9.2±20.8 | 16.2±27.0 | 22.0±31.2 | <0.001 |
| PRA2 | 9.2±21.5 | 15.2±28.0 | 23.3±32.7 | <0.001 |
| **Pre-transplant DSA** | 249 (6.8) | 89 (19.1) | 143 (31.3) | <0.001 |
| Missing | 1325 (36.3) | 147 (31.5) | 104 (22.8) |  |
| **Calcineurin inhibitor** |  |  |  |  |
| Tacrolimus | 3524 (96.4) | 456 (97.6) | 454 (99.3) | 0.002 |
| Cyclosporine | 117 (3.2) | 9 (1.9) | 2 (0.4) | 0.002 |
| **Donor characteristics** | | | | |
| **Age**, years | 47.1±12.8 | 48.9±13.4 | 47.7±13.4 | 0.018 |
| **Female sex** | 1846 (50.5) | 183 (39.2) | 170 (37.2) | <0.001 |
| **Body mass index**, kg/m^2^ | 23.9±3.3 | 24.0±3.5 | 23.8±3.6 | 0.521 |
| **Relation to the recipient** |  |  |  | <0.001 |
| Deceased donor | 1193 (32.6) | 269 (57.6) | 247 (54.1) |  |
| Living, related | 1434 (39.2) | 94 (20.1) | 113 (24.7) |  |
| Living, unrelated | 1028 (28.1) | 104 (22.3) | 97 (21.2) |  |
| **CRRT** | 51 (1.4) | 34 (7.3) | 18 (3.9) | <0.001 |
| Missing | 2528 (69.2) | 207 (44.3) | 218 (47.7) |  |
| **Current smoker** | 2452 (67.1) | 270 (57.8) | 270 (59.1) | <0.001 |
| **Hypertension** | 515 (14.1) | 94 (20.1) | 92 (20.1) | <0.001 |
| **Serum creatinine**, mg/dL | 0.95±0.70 | 1.41±1.32 | 1.46±1.39 | <0.001 |
| **Cold ischemic time** |  |  |  | <0.001 |
| 0-6 hours | 2812 (76.9) | 341 (73.0) | 316 (69.2) |  |
| 6-12 hours | 192 (5.3) | 47 (10.1) | 41 (9.0) |  |
| > 12 hours | 6 (0.2) | 5 (1.1) | 2 (0.4) |  |
| Missing | 645 (17.7) | 74 (15.9) | 98 (21.4) |  |

Values are mean±standard deviation or n (%).

ESRD, end-stage renal disease; PCKD, polycystic kidney disease; PRA, panel reactive antibody; DSA, donor-specific antigen; CRRT, continuous renal replacement therapy
